# Unilateral curved versus bipedicular vertebroplasty in the treatment of osteoporotic vertebral compression fractures

**DOI:** 10.1186/s12893-019-0653-y

**Published:** 2019-12-12

**Authors:** Rui Zhong, Jianheng Liu, Runsheng Wang, Yihao Liu, Binbin Chen, Wei Jiang, Keya Mao, Peifu Tang

**Affiliations:** 1 Department of Orthopaedics, Chinese PLA General Hospital, Beijing, 100853 China; 2 Sports Hospital Affiliated to Chengdu Sport Institute, Chengdu, 610041 Sichuan China

**Keywords:** Osteoporotic vertebral compression fractures, Curved approach, Bipedicular, Vertebroplasty, Cement leakage, X-ray exposure

## Abstract

**Background:**

Vertebral compression fracture is one of the most common complications of osteoporosis. In this study an unilateral curved vertebroplasty device was developed, and the safety, effectiveness, and surgical parameters of curved vertebroplasty (CVP) in the treatment of painful osteoporotic vertebral compression fractures was investigated and compared with traditional bipedicular vertebroplasty (BVP).

**Methods:**

We investigated 104 vertebral augmentation procedures performed over 36 months. CVP and BVP procedures were compared for baseline clinical variables, pain relief (Visual Analog Scale, VAS), disability improvement (Oswestry Disability Index, ODI), operation time, number of fluoroscopic images, volume of cement per level, and cement leakage rate for each level treated. Complications and refracture incidence were also recorded in the two groups.

**Results:**

The VAS and ODI in both group had no significant difference preoperative (*P* > 0.05), and a significant postoperative improvement in the VAS scores and ODI was found in both group (*P* < 0.001). However, the CVP group had significantly lower operation time, number of fluoroscopic images, and cement leakage rate per level than the BVP group (*P* < 0.05); however, the volumes of cement per level were similar in the two groups (P > 0.05). Neither group had any serious complications. Five and two patients in the BVP group developed refractures at non-adjacent and adjacent levels, respectively, with one patient developing refractures twice; however, none of the patients in the CVP group developed refractures at any level.

**Conclusions:**

Our findings revealed that both CVP and BVP were safe and effective treatments for osteoporotic vertebral compression fractures, and CVP entails a shorter operation time, less exposure to fluoroscopy, and lower rate of cement leakage.

## Background

Osteoporosis is a systemic disorder characterized by low bone mass and microarchitectural deterioration of bone tissue, with a consequent increase in bone fragility and susceptibility to fractures [1]. The most common type of fractures due to the bone fragility associated with osteoporosis are vertebral compression fractures (OVCFs), affecting 25% of postmenopausal women and more than 200 million individuals worldwide [2]. OVCFs are known to cause substantial pain and deformity, which in turn leads to disability and reduced quality of life and significantly increases the lifetime risk of fractures [3].

The evidence-based clinical practice guidelines put forth by the American Academy of Orthopedic Surgeons in 2011 recommend against vertebroplasty for osteoporotic VCF patients [4]. However, two very recent systematic reviews have shown that percutaneous vertebral augmentation (PVA) affords better outcomes in terms of pain relief, functional recovery, and health-related quality of life as compared to non-operative or sham treatment in cases of painful OVCF [5, 6]. Furthermore, PVA is associated with lower risk of mortality than non-surgical treatments among Medicare beneficiaries [7, 8].

The most common PVA methods currently used in the clinic are balloon kyphoplasty and vertebroplasty. Cement leakage is the most important complication of PVA, especially vertebroplasty, mainly because it involves a high-pressure injection. Traditionally, the standard technique for PVA is a bipedicular approach [9]. Subsequently, a unipedicular approach was developed, and it has been shown to be associated with a lower operating time, extent of trauma, and X-ray exposure as well as better cost effectiveness [10, 11]. However, with the unipedicular approach, the cement is likely to remain in the same side of application, and this non-uniform distribution of bone cement may increase the risk of re-collapse of the non-augmented side, especially during lateral bending. Furthermore, the unipedicular approach requires a more aggressive, lateral-to-medial approach as compared to the bipedicular approach, which increases the risk of inadvertent injury to paravertebral vessels or nerves.

**Fig. 1:**
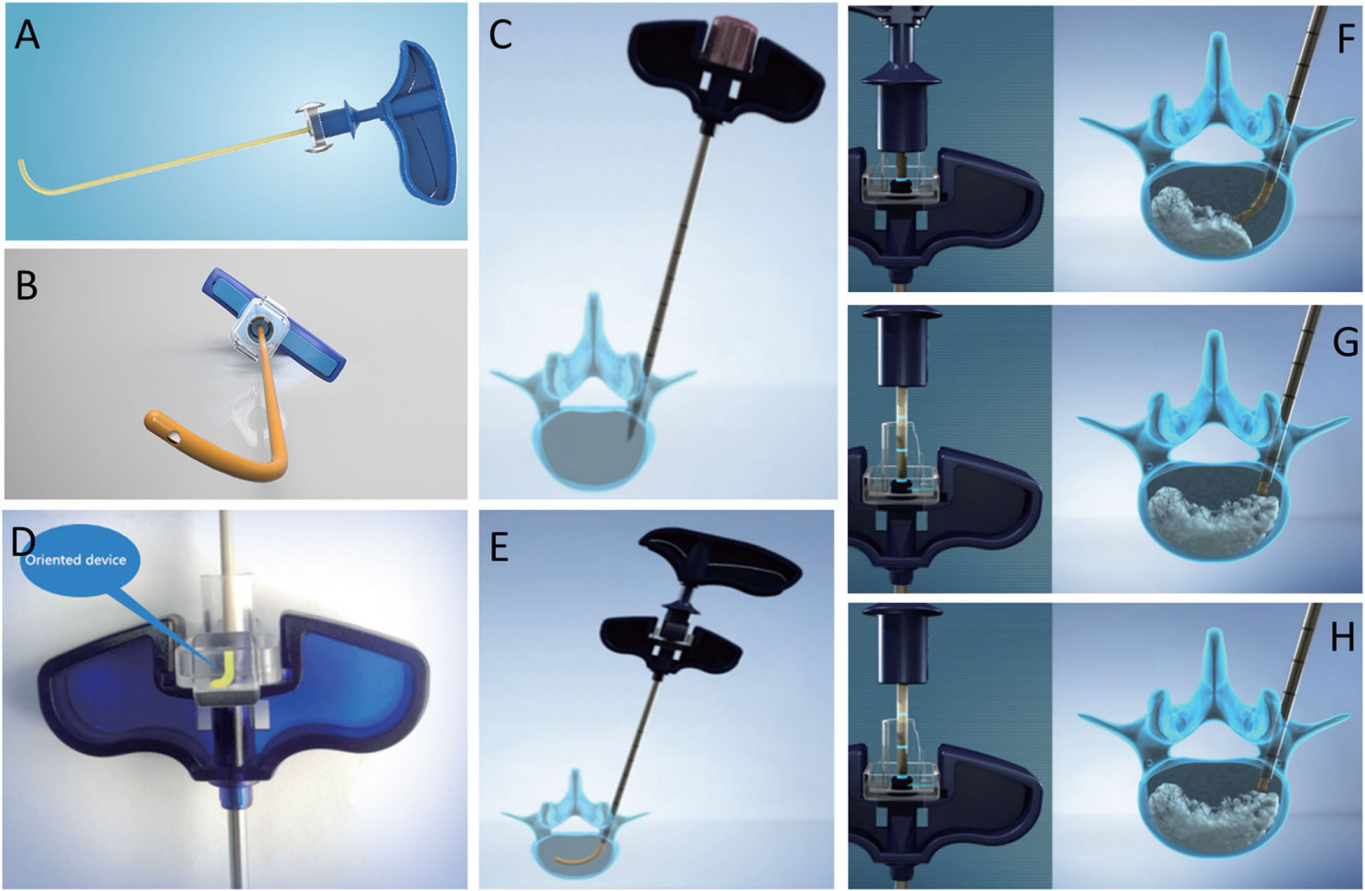
Percutaneous Curved Vertebroplasty Device **a**, Standard transpedicular approach; **b**, insert curved injection cannula via straight trocar into the contralateral hemivertebra body; **c**, orientation of the device avoids cannula access in the wrong position; **d**, **e**, The veutro design of the side opening near the tip of the curved cannula prevents distribution of cement into the posterior border of the vertebra; **f**, **g**, **h**, The cannula was withdrawn point-by-point and the bone cement (1–2 mL) was injected with a specially designed delivery at each point

Thus, the ideal technology would involve the achievement of uniform bone cement distribution within the vertebral body with the use of the unipedicular approach. Our team designed a device named percutaneous curved vertebroplasty device (percutaneous CVP device, manufactured by Hicren, Ningbo, China). This device uses a curved injection cannula via a straight trocar introducer to augment the two sides of the vertebral body by means of the unipedicular approach. Low-pressure cement injection at multiple points theoretically reduces the leakage rate (Fig. 1a-h). A similar type of device with a curved needle [12–14] (Avaflex; Cardinal Health, Dublin, Ohio) is used to evaluate the safety and effectiveness of the curved needle in comparison with non-curved needle techniques. This CVP device has been used in treating painful OVCF in the department of orthopeadics at our hospital since July 2013.

In this study, the safety and effectiveness of the CVP technique were evaluated in comparison with the traditional BVP technique, with both being applied during the same study period. Additionally, we sought to assess the differences between the two procedures in terms of operation time, number of fluoroscopic images, incidence of cement leakage, and incidence of vertebral refracture.

## Methods

### Patient selection

This study was designed as a retrospective investigation of 104 patients who underwent surgical correction of OVCF at our institution. The study protocol was approved by Ethics Committee of Chinese People’s Liberation Army general hospital. Written informed consent was obtained from each patient prior to the study.

Patients were identified through the electronic medical record system maintained at our center, and all the imaging data (include preoperative, postoperative, and follow-up studies) were collected and saved onto our hard disk. Data were independently collected and sorted by two surgeons who were not involved in the operation, and the collected data were crosschecked. All the enrolled patients had failed to respond to conservative medical therapy.

The inclusion criteria for the study were as follows: (1) age above 50 years; (2) OVCFs at no more than two levels, with both pedicles intact; (3) less than 50% collapse of the vertebral body, as determined by lateral plain radiographs; (4) focal back pain in the midline without any neurologic deficits; and (5) back pain in the region corresponding to the location of the OVCFs, as determined by magnetic resonance imaging (MRI). Patients were excluded from this study if they met the following criteria: (1) vertebral compression fracture due to causes other than osteoporosis; (2) spinal cord compression or stenosis of the vertebral canal at a degree of > 30% of the local canal diameter; (3) serious vertebral fracture with posterior ligament complex injury; (4) history of incurable bleeding disorders; (5) history of systemic or local spine infections; (6) severe comorbid disease that can lead to intolerance of the surgery; and (7) history of allergy to the PVA instruments or bone cement.

Between July 2013 and June 2016, 110 patients underwent 118 vertebral augmentation procedures: three of them underwent balloon kyphoplasty; two patients underwent three-segment augmentation, and one patient each underwent four-segment and five-segment augmentation. One hundred and three patients underwent 111 vertebroplasty procedures at 127 levels. Two patients died due to complications unrelated to the procedures, whereas five were lost to follow-up within 12 months of the operation; these patients were excluded from the study.

**Fig. 2:**
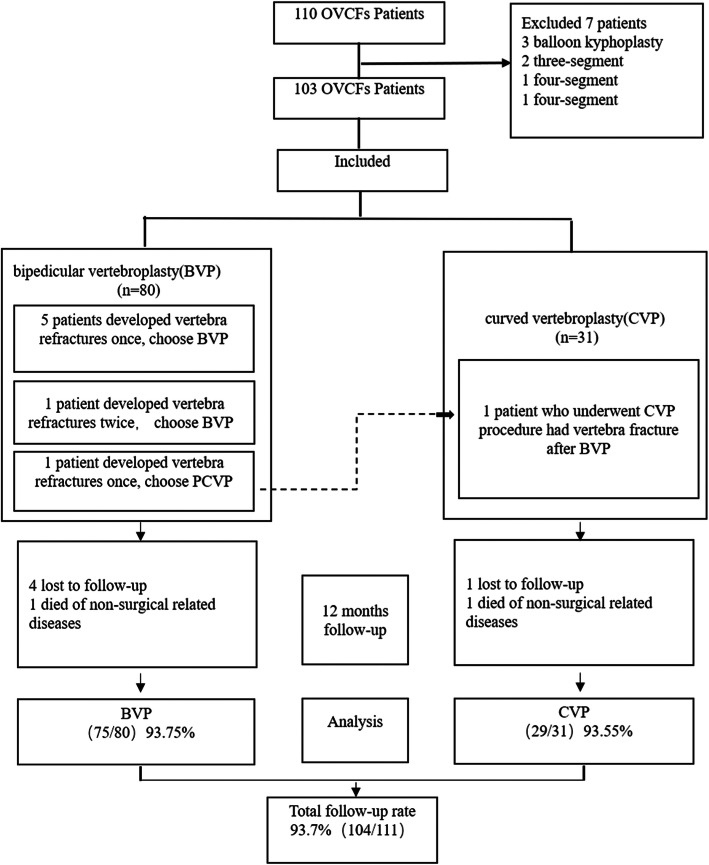
Flow chart for the study The number of cases is calculated by number of procedures

Finally, 96 patients who underwent a total of 104 vertebral augmentation procedures at 117 levels were included in this study, and the follow-up rate was 93.7% (104/111). Twenty-nine patients (3 male and 26 female; average age, 70.7 ± 7.5 years) underwent 29 CVP procedures, with 35 OVCFs. One patient who first underwent BVP subsequently developed nonadjacent refractures, for which CVP was performed. Further, 68 patients (12 male and 56 female; average age, 73.8 ± 8.2 years) underwent 75 BVP procedures with 82 OVCFs. The body mass index (BMI) in the CVP and BVP groups was 25.1 ± 5.0 and 24.0 ± 3.0, respectively. In the CVP group, 23 patients underwent single-level procedures, while 6 underwent two-level procedures; in the BVP group, the corresponding numbers were 68 and 7, respectively. The majority of the vertebral fractures were in the thoracolumbar region, in both groups. Moreover, there were no significant differences between the two cohorts in terms of demographic characteristics, gender ratio, BMI, number of fractures per patient, time from injury, and vertebral region treated (Table 1, Fig. 2).

**Table 1:** Pre-operative demographic data of patients undergoing either CVP or BVP

Variable	CVP group *N* = 29	BVP group *N* = 75	*P*-value
Mean age (year)	70.7 ± 7.5	73.8 ± 8.2	0.082
Male/Female	3/26	12/63	0.550
BMI	25.1 ± 5.0	24.0 ± 3.0	0.196
Fracture per patient			
One-level/Two-level	23/6	68/7	0.182
Mean time from			
Injury (day)	19.1 ± 25.3	28.7 ± 39.4	0.229
Vertebral region treated			
Thoracic (T1–10)	2 (5.7%)	5 (6.1%)	0.920
Thoracolumbar (T11-L2)	30 (85.7%)	68 (82.9%)	
Lumbar (L3–5)	3 (8.6%)	9 (11.0%)

### Surgical procedures

All the procedures were performed by the same operation team. All team members were senior orthopedists specializing in spine surgery. The entire procedure was completed under local anesthesia, with the patient placed in the prone position on a radiolucent operating table. All the procedures were guided by G-arm fluoroscopy.

**Fig. 3:**
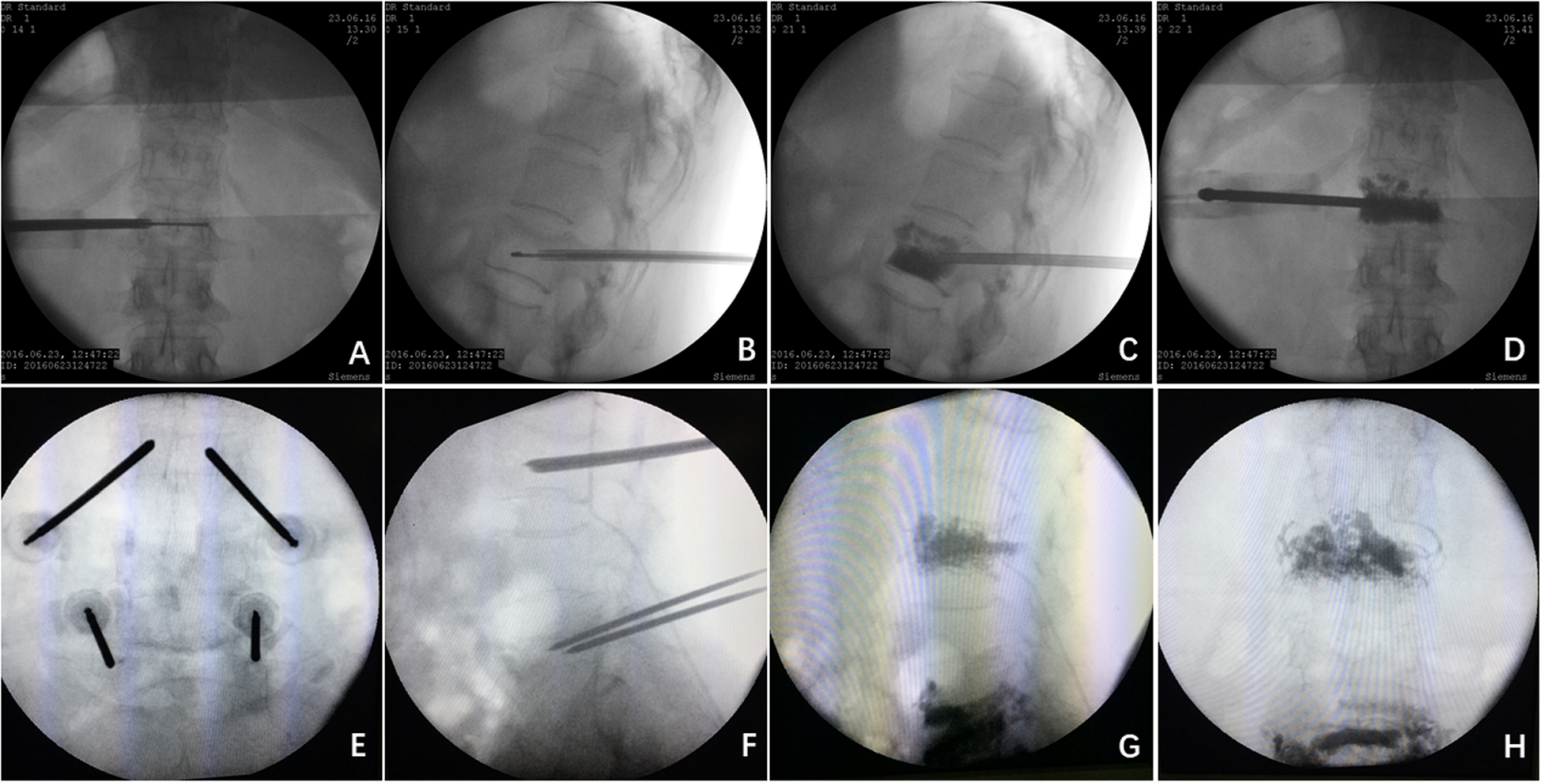
Injection procedure comparison between curved and bipedicular approach vertebroplasty **a**, **b**, **c**, **d**: CVP group after the introducer was accessed into optimal position, the curved injection cannula is inserted, anteroposterior fluoroscopy the tip cross the midline, and lateral fluoroscopy the tip in the forepart of the vertebra body, cement was distribute in both side of the vertebra body. **e**, **f**, **g**, **h**: BVP group the two straight introducer were accessed into 1/3 forepart of the vertebra body in the lateral fluoroscopy, cement distribute in both side of the vertebra body while leaking into the intervertebral space

CVP group: The device used was designed by our team and manufactured by Hicren, Ningbo, China. After making the initial incisions, an external introducer (3.0 mm in diameter) was advanced with a coaxial puncture by using the standard transpedicular approach. The introducer was targeted by using a standard technique modified to place its tip in the posterior one-fourth part of vertebral body. After withdrawing the inner puncture needle, an orientation device was placed with the arrow in front of the handle, and the curved injection cannula was advanced through this introducer. The curved injection cannula was then inserted to access the contralateral hemivertebra body. With the fluoroscope held in the optimal position (for anteroposterior fluoroscopy, the tip crossed the midline; for lateral fluoroscopy, the tip was placed in the middle of the vertebral body), the inner nitinol needle was withdrawn, and the outer polyether-ether-ketone injection cannula with side openings near the tip was retained. The cannula was withdrawn point-by-point, and bone cement (1–2 mL) was injected with a specially designed delivery at each point. The injection procedure was carefully monitored under close lateral fluoroscopy (Fig. 3 a-d).

BVP group: After the incisions were made, two external introducers (2.5 mm in diameter) with coaxial punctures were advanced using a standard transpedicular approach and placed on both sides of the middle and anterior one-thirds of the injured vertebral body under fluoroscopic guidance. Once the needles were held in the optimal position, polymethylmethacrylate (PMMA) bone cement was injected via a specially designed delivery system (Stryker, USA). The injection procedure was closely monitored under strict lateral fluoroscopic guidance and stopped once the vertebra was filled well or when the cement reached the dorsal quarter of the vertebral body (Fig. 3 e-h).

The patients were recommended bed rest for at least 24 h after the procedure and advised to resume functional activities with the help of a spinal protector. Additionally, systemic anti-osteoporosis therapy was administered.

### Outcome examinations

The safety indices include incidence rates of nerve and blood vessel injury and complications. Serious complications that occurred either during or after the operation were recorded. Asymptomatic polymethylmethacrylate leaks were not considered as complications. Subsequent vertebral body fractures of the operated vertebrae or refractures at adjacent or non-adjacent levels during the follow up period were also recorded. We only recorded fragility vertebral fractures (definite diagnosis established by MRI) and excluded burst fractures due to high-energy trauma.

The clinical outcomes were evaluated in terms of analgesic efficacy by comparing the data of the VAS scores recorded before the operation as well as one day, three months, six months and twelve months after the operation; the outcomes were also evaluated in terms of functionality by comparing the values of the Oswestry Disability Index (ODI) recorded before the operation as well as three months, six months and twelve months after the operation.

The following surgical parameters were also assessed: (1) operation time (time from skin incision to suturing, excluding the time taken for checking the preoperative index levels, with two surgeons cooperating with each other to restrict the operation time as much as possible), (2) number of fluoroscopic images, including intermittent anteroposterior and lateral images taken before the start of the operation up to the end of the procedure, (3) volume of cement per level, and (4) the incidence of cement leakage from the vertebral body, as determined by post-operative radiography or computed tomography (In order to reduce unnecessary radiation exposure in patients, patients with obvious leakage into the intervertebral space or paravertebral space were not required to undergo CT scans. CT scans were only acquired in cases where the cement was close to the posterior wall of the vertebral body, which increases the risk of leakage into the spinal canal or basivertebral veins. The classification of cement leakage was based on previous guidelines [15].

### Statistical analysis

Data are presented as the mean ± standard deviation. SPSS for Windows Version 19.0 (SPSS, Chicago, IL) was used for the analysis. The statistical significance of the inter- and intragroup differences in age, BMI, time from injury, operation duration, number of fluoroscopic images, and volume of cement were evaluated using the independent *t*-test. The inter- and intra-group differences between the pre- and post-surgical VAS and ODI data were evaluated using the paired-samples *t*-test and independent *t*-test. Differences in the gender ratio, fracture per patient, vertebral region treated, and cement leakage between the two groups were assessed using the χ^2^ test. The level of statistical significance was set at *P* < 0.05.

## Results

### Safety

No puncture-related complications were noted, either during or after the operation in both groups. In the BVP procedures, intraspinal cement leakage was detected: two of these cases were asymptomatic and detected only on postoperative CT scans. In the reaming case, cement leakage occurred during the L1 and L2 OVCF procedure, with the leakage extending into the intraspinal space, as determined by intraoperative fluoroscopy. The cement which leakage into spinal canal was immediately removed through percutaneous transforaminal endoscopy, thereby preventing nerve injury.

### Clinical outcomes

There were no differences between the CVP and BVP groups in terms of the preoperative VAS scores (*P* = 0.626) and ODI (*P* = 0.529). However, both groups showed statistically significant differences in the pre- and postoperative scores of VAS (*P* = 0.000) and ODI (*P* = 0.000), indicating significant improvement in pain and functionality after the operation. Further, the VAS scores and ODI at day one (VAS, *P* = 0.691), 3 months (VAS, *P* = 0.104 and ODI, *P* = 0.288), and 6 months (VAS, *P* = 0.222 and ODI, *P* = 0.386) after the operation did not show any difference between the 2 groups, indicating no difference in terms of the clinical outcomes (Table 2).

**Table 2:** Preoperative and postoperative VAS and ODI of CVP and BVP groups

Variable	CVP group N = 29	BVP group N = 75	P-value
Preoperative VAS	8.3 ± 1.0	8.2 ± 0.8	0.626
Postoperative 1 day VAS	3.2 ± 1.7#	3.4 ± 1.2#	0.691
Postoperative 3 month VAS	2.4 ± 1.2#	2.8 ± 0.9#	0.104
Postoperative 6 month VAS	2.0 ± 1.1#	2.3 ± 1.0#	0.222
Postoperative 12 month VAS	1.9 ± 1.0#	1.8 ± 0.9#	0.760
Preoperative ODI	75.0 ± 11.1	73.4 ± 11.7	0.529
Postoperative 3 month ODI	42.5 ± 15.1*	45.5 ± 11.4*	0.288
Postoperative 6 month ODI	36.2 ± 10.4*	38.0 ± 9.6*	0.386
Postoperative 12 month ODI	28.7 ± 11.5*	29.3 ± 10.3*	0.797

The table shows results of the outcome measures for both groups at preoperative, 3 months, 6 months and 12 months postoperative. Data are expressed as mean values (SD). Visual Analogue Scale (VAS, scale: 0 to 10), Oswestry Disability Index (ODI, scale: 0–100).# compare with preoperative VAS, *P*<0.05; * compare with preoperative ODI, *P*<0.05

### Surgical parameters

The mean operation time in the CVP group was 29.2 ± 8.0 min, which was less than that in the BVP group (41.0 ± 8.2 min; *P* < 0.001). The number of fluoroscopic images acquired in the CVP and BVP group was 18.9 ± 3.8 and 25.9 ± 4.8 (P < 0.001), respectively (Table 3). The volume of cement per level was 4.5 ± 2.1 mL, 5.3 ± 1.4 mL, and 4.3 ± 1.5 mL in the thoracic, thoracolumbar, and lumbar regions in the CVP group, while those in the BVP group were 4.2 ± 2.2 mL, 5.5 ± 1.8 mL, 6.5 ± 2.0 mL, respectively, indicating no significant difference between the two (*P* = 0.875, *P* = 0.725, *P* = 0.120). The cement leakage rate in the CVP group was 22.9% (8 of 35), which was significantly less than that in the BVP group (43.9%; 36 in 82; *P* = 0.038; Table 3). In the CVP group, cement leakage extended to the periphery of the vertebral body in 2 cases, the disc space in 3 cases, and the paraspinal tissues in 3 cases; the corresponding numbers of cases were 10, 14, and 9 in the BVP group, with an additional 3 cases showing intraspinal leakage. No nerve damage occurred in either group.

**Table 3:** Surgical parameters in the two groups

Variable	CVP group	BVP group	*P*-value
	N = 29	N = 75	
Mean (SD) Operation duration (min)	29.2 ± 8.0	41.0 ± 8.2	0.000*
Duration of fluoroscopy	18.9 ± 3.8	25.9 ± 4.8	0.000*
Volume of cement per level (mL)			
T(T1–10)	4.5 ± 2.1	4.2 ± 2.2	0.875
TL(T11-L2)	5.3 ± 1.4	5.5 ± 1.8	0.725
L(L3–5)	4.3 ± 1.5	6.5 ± 2.0	0.120
Cement leakage rate per level treated (n%)	8/35 (22.9%)	36/82 (43.9%)	0.038*

*P < 0.05

### Refracture occurrence rate

During the study period, five and two patients developed fragile vertebral refractures at nonadjacent and adjacent levels, respectively (one patient developed adjacent level refractures twice), in the BVP group. In contrast, none of the patients developed refractures at either nonadjacent or adjacent levels in the CVP group during the 36-month period. Further, none of the patients in the study developed refractures at the operated level.

## Discussion

The standard technique for PVA involves a bipedicular approach. It has been shown that compared to the the bipedicular approach, the unipedicular approach entails less operation time, trauma, and exposure to X rays as well as better cost effectiveness [10, 11]. In the unipedicular approach, although the cement is not distributed to the contralateral aspect of the vertebral body, many in vitro biomechanical tests have shown that when cement augmentation crosses the midline, stiffness increases on both sides [16–18]. However, these studies were only focused on axial mechanics, which cannot simulate the complex mechanisms involved in thoracolumbar motion. This uneven distribution of bone cement increases the risk of recollapse on the contralateral side, especially during lateral bending. Furthermore, if distribution of the cement across the midline is to be attempted, the traditional, straight-needle unipedicular approach, which requires a more aggressive lateral-to-medial approach or, in some cases, an extrapedicular approach that may put paravertebral vessels or nerves at risk. In CVP, a curved cannula is used via a standard transpedicular approach, which allows for easy distribution of the cement on the contralateral hemivertebral side; this lowers the risk of injury to paravertebral vessels or nerves, to a level equal to that afforded by the BVP approach. In our study, no puncture-related complications occurred in any of the groups, either during or after the operation.

No significant differences were noted between the two cohorts regarding age and gender, BMI, and time from injury to operation. The preoperative VAS score and ODI did not differ in the two groups. Compared to the traditional BVP procedure, the CVP procedure afforded significant relief in terms of focal back pain and disability improvement, with the beneficial effects persisting for more than 12 months. This indicates that CVP is as effective as traditional vertebral augmentation techniques in treating OVCFs using the curved needle [12–14].

Vertebroplasty via the bipedicular approach can re-establish the biomechanical balance between the two sides [9]; theoretically, the risk of bone cement leakage in the bipedicular approach is also twice that in the unipedicular approach [19]. High fracture severity grade, cement viscosity, and volume of bone cement are the strongest independent risk factors for bone cement leakage after PVP [20, 21]; in our study, there were no differences between the CVP and BVP groups with respect to these three parameters. In both groups, excessive efforts to distribute cement across the whole vertebral body were not made, but reasonable distribution of bone cement was achieved to an extent sufficient enough to alleviate pain and restore the biomechanics of the vertebrae. The volumes of the bone cement used in both groups were within the reasonable range, but significant differences were noted in leakage rate per level treated. The desired radiographic endpoint in PVP is bilateral cement deposition, and the major obstacle to achieving this endpoint is unwanted cement migration toward the posterior vertebral body cortex or cement leakage into the paravertebral or basivertebral veins. Aggressive cement deposition from a fixed needle position may necessitate the application of increased pressure, which can lead to cement leakage. In CVP, the curved injection cannula can easily access the contralateral hemivertebral body; the cement is delivered into the vertebral body through the side openings in the ventral tip of the cannula because of which it is not likely to spread to the posterior portion of the vertebral body. The cannula was withdrawn point-by-point, and bone cement (1–2 mL) was injected at each point. Compared with a single high-pressure delivery, low-pressure delivery at multiple points allows for adequate distribution of the cement, while also minimizing the risk of intra-spinal or intravenous leakage. This also allows for the even distribution of bone cement in the vertebral body space in the CVP group, unlike the localized distribution of the cement around the puncture channel in the BVP group. The leakage rate in the CVP group was significantly lower than that in the BVP group, and even lower than that reported in the literature [15, 20, 22]. Additionally, CVP reduces puncture trauma on one side and remains simple as the traditional unipedicular PVP. With no more complicated procedures addition, the CVP group guarantees reductions of operation time and the number of fluoroscopic images, and consequently outperforms the BVP group. The reduced radiation exposure further reduce health risks posed to medical staff and patients throughout the operation procedure. Notably, CVP is more suitable for the aged, specifically for patients who cannot bear lying in the prone position for long periods. The deformation resistance of the curved nickel-titanium alloy core stays between 35 and 45 N, allowing a smooth entering and withdraw from the straight introducer without imposing great effort by the operator. However, it is also due to the relatively rigid range of deformation, the curved core may not be able to insert into the vertebral body once the bone is too hard and CVP can only be used for the treatment of the OVCFs.

None of the patients developed refractures at nonadjacent or adjacent levels in the CVP group, whereas five and two patients in the BVP group developed refractures at nonadjacent and adjacent levels (one patient with adjacent level refractures occurring twice), respectively, during the 36-month period. None of the operated vertebra in both groups developed refractures; this may be attributed to the even and adequate distribution of bone cement. Low bone mineral density and trauma are the main risk factors for vertebral refracture. The BVP group had seven cases of refracture, with two occurring at adjacent levels; however, due to the fewer number of cases in the CVP group, it cannot be concluded whether CVP reduces the risk of refracture at adjacent levels.

In cases of multiple OVCFs, the procedure is complex and precise preoperative planning is necessary to determine the surgical approach; the combined application of several techniques may help resolve the issue. Therefore, in our study, we included cases with corrections required only at one or two levels. Additionally, since the scope of kyphosis correction and restoration of the vertebral body height is limited, we included only cases with vertebral body collapse of less than 50%, on lateral plain radiographs. For cases with collapse beyond 50% and severe kyphosis, we recommend balloon kyphoplasty to correct kyphosis and decrease the cement leakage rate. We have attempted to apply our “curved” device in balloon kyphoplasty, and although the instrument is under development and initial testing, we hope that this approach will be valuable in clinical practice in the future.

This study does have some limitations. For those with less than 50% compression degree painful OVCFs, we choose PVP. The specific approach choice is made by the patient after a full explanation of the therapeutic procedure. There is a risk for selection bias, but the bias is likely balanced between the study cohorts. Since this is a retrospective design, despite a rigorous search through electronic medical records and patients’ fluoroscopy image, observation bias cannot be ruled out. Furthermore, the follow-up investigation was only set at three, six and twelve month after surgery; longer follow-up durations might show differences between the groups. All patients did not undergo preoperative bone mineral density (BMD) measurement; therefore, we did not include this index in our study. BMD may indicate the risk of refracture; however, this bias may be equal in both groups.

## Conclusion

We have designed a device named percutaneous curved vertebroplasty device in this clinical study. This device uses a curved injection cannula via a straight trocar introducer to augment the two sides of the vertebral body by means of the unipedicular approach. And we also found that CVP is as safe and effective as BVP in terms of improvement in pain and function in OVCFs, and CVP offers the additional advantages of shorter operation time, less exposure to fluoroscopy, and lower rate of cement.

## Data Availability

The datasets during and/or analyzed during the current study are available from the corresponding author on reasonable request.

## Abbreviations

BVP: Bipedicular vertebroplasty
CVP: Curved vertebroplasty
ODI: Oswestry disability index
OVCFs: Osteoporosis are vertebral compression fractures
PVA: Percutaneous vertebral augmentation
VAS: Visual analog scale
